# Population Genomics Insights into the First Wave of COVID-19

**DOI:** 10.3390/life11020129

**Published:** 2021-02-07

**Authors:** Maria Vasilarou, Nikolaos Alachiotis, Joanna Garefalaki, Apostolos Beloukas, Pavlos Pavlidis

**Affiliations:** 1 Foundation for Research and Technology Hellas (FORTH) and Department of Biology, Institute of Molecular Biology and Biotechnology (IMBB), University of Crete, 70013 Crete, Greece; maria_vasilarou@imbb.forth.gr; 2 Faculty of EEMCS, University of Twente, 7522NB Enschede, The Netherlands; n.alachiotis@utwente.nl; 3 Institute of Computer Science (ICS), Foundation for Research and Technology Hellas (FORTH), 70013 Heraklion, Greece; igarefalaki1390@gmail.com; 4 Department of Biomedical Sciences, University of West Attica, 12243 Athens, Greece; 5 Institute of Infection and Global Health, University of Liverpool, Liverpool L69 7BE, UK

**Keywords:** SARS-CoV-2, population genetics, recombination, mutation rate, selective sweeps, demographic inference

## Abstract

Full-genome-sequence computational analyses of the SARS-coronavirus (CoV)-2 genomes allow us to understand the evolutionary events and adaptability mechanisms. We used population genetics analyses on human SARS-CoV-2 genomes available on 2 April 2020 to infer the mutation rate and plausible recombination events between the Betacoronavirus genomes in nonhuman hosts that may have contributed to the evolution of SARS-CoV-2. Furthermore, we localized the targets of recent and strong, positive selection during the first pandemic wave. The genomic regions that appear to be under positive selection are largely co-localized with regions in which recombination from nonhuman hosts took place. Our results suggest that the pangolin coronavirus genome may have contributed to the SARS-CoV-2 genome by recombination with the bat coronavirus genome. However, we find evidence for additional recombination events that involve coronavirus genomes from other hosts, i.e., hedgehogs and sparrows. We further infer that recombination may have recently occurred within human hosts. Finally, we estimate the parameters of a demographic scenario involving an exponential growth of the size of the SARS-CoV-2 populations that have infected European, Asian, and Northern American cohorts, and we demonstrate that a rapid exponential growth in population size from the first wave can support the observed polymorphism patterns in SARS-CoV-2 genomes.

## 1. Introduction

In late December 2019, Chinese health authorities reported a cluster of atypical pneumonia cases epidemiologically linked with the Huanan Seafood Wholesale Market in Wuhan, Hubei Province, China [1]. On 7 January 2020, these cases were associated with a novel human coronavirus (hCoV), dubbed SARS-coronavirus (CoV)-2 [2], which constitutes the third documented spillover from mammals, but it is divergent from SARS-CoV and MERS-CoV that caused past epidemics [3,4]. Two weeks later, the United States of America reported the first confirmed case of SARS-CoV-2 infection while the first three cases in Europe were confirmed on 24 January 2020 [5]. As of 6 December, the pandemic coronavirus-associated acute respiratory disease called coronavirus disease 19 (COVID-19) has infected more than 65.8 million people and has caused more than 1.5 million deaths (WHO, COVID-19 Situation Report 6 December 2020). 

As the outbreak progresses over time, laboratories around the world are sequencing SARS-CoV-2 genomes from various human sources and timepoints to track the dispersal pattern of the pandemic. SARS-CoV-2 belongs to the Betacoronavirus genera and, structurally, is an enveloped RNA virus with a non-segmented, positive-sense (+ssRNA) genome of ≈30 kb, amongst the largest identified RNA genomes [6,7]. The application of next-generation sequencing (NGS) approaches on pathogens can elucidate important features such as disease transmission and virulence [8]. In fact, NGS data accumulate at an unprecedented rate, and during the first wave of the COVID-19 epidemic, the Global Initiative on Sharing All Influenza Data (GISAID) database, which originally promoted the international sharing of all influenza virus sequences, as of 2 April, included 3230 SARS-CoV-2 genome data submissions (2305 full-length sequences with high-coverage).

Population genetics can provide insights into the spread and epidemics of viruses because it offers the machinery to estimate the values of parameters such as the mutation and recombination rate. Both of these parameters are important for the evolution and the management of viral diseases. Mutation rate and recombination as well as stochastic (random genetic drift) and non-stochastic processes (selection) are the dominant forces that shape viral diversity in natural populations of RNA viruses. RNA viruses demonstrate the highest mutation rates of any group of organisms [9], which may lead to viral adaptation to selective pressures and may alter their virulence. In addition, recombination has an important role in evolution because it elevates haplotypic variability in viral populations and potentially generates highly fitted genomes more rapidly than by mutation rate alone, especially when epistatic interactions are present between different genomic locations. Research on viral recombination rates has demonstrated its association with increased genetic diversity and the generation of novel lineages and new pathogenic recombinant circulating viruses [10,11]. Many recent studies suggest that recombination events may have shaped the architecture of the SARS-CoV-2 genome [12,13]. It has been suggested that it shares 96.3% genetic similarity with a bat coronavirus (CoV) [14] and 91.2% genetic similarity with two pangolin coronavirus (CoV) genomes, especially in the S gene. Furthermore, the latest studies suggest that the pandemic SARS-CoV-2 genome might have evolved from a bat CoV [6,13,15,16,17,18,19,20]. Recombination events between SARS-CoV-2 and RaTG13 (bat CoV) have also been observed in ORF1a [1], in the receptor-binding domain (RBD), and at the furin site [21]. More recent findings suggest that the SARS-CoV-2 genome backbone evolved from Yunnan bat virus-like SARs-CoVs, and its RBD region acquired from pangolin virus-like SARS-CoVs [17,22,23].

High mutation and/or recombination rates imply that developing a successful vaccine will be challenging since viral genomes will be able to adapt fast in human interventions [6]. Moreover, population genetics provides tools to infer the recent evolutionary history of populations, both adaptive and non-adaptive. Thus, by using statistical approaches, such as the approximate Bayesian computation (ABC) [24], it is feasible to assess past population size changes by using only present-day genomic data. RNA viruses demonstrate immense population sizes [9], and the study of the population demographic history could support viral epidemiological research. A previous study, in particular, has associated the change from a bat CoV population of constant size to a population growth of CoVs from other hosts, with the interspecies transmission of viruses from their original reservoir to an alternate host [25]. Furthermore, selective sweep theory [26,27,28] allows for the localization of the targets of recent and strong selection on genomes.

In this study, we aim to elucidate the evolution of early viral strains by implementing population genetics approaches on full-length human SARS-CoV-2 genomes available from the GISAID database [29]. Understanding the SARS-CoV-2 evolutionary processes would enable us to assess critical parameters, such as the mutation rate and plausible recombination as well as recent selective sweeps and population demographic parameters, which can directly affect the evolution of this virus within the human population. Such results will support epidemiological research on tracing dispersal patterns of the pandemic and on designing therapeutic or preventive strategies.

## 2. Materials and Methods

Our dataset comprised all publicly available genomic data from the GISAID database, downloaded on 2 April 2020, to infer evolution of SARS-CoV-2 strains circulated during the first wave of the pandemic. The initial dataset comprised 3230 full genome assembled sequences, with only 2305 of those fulfilling the high-coverage criterion. We applied additional filters to keep only high-quality sequences (see below). The final dataset consists of 1895 human SARS-CoV-2 genomes and two outgroup sequences: bat CoV and pangolin CoV genomes. All analyses except for selective-sweep localization were performed on this dataset.

### 2.1. Mutation Rate Analysis and Estimation of the Time of the Most Recent Common Ancestor between Bat CoV and SARS-CoV-2

We performed a regression analysis to estimate the mutation rate per nucleotide and per day, denoted by *μ*. The collection date of the samples was recorded in the GISAID repository. Let *h_i_* be the collection date for sample *i*. Divergence between sample *i* and the bat CoV sequence was estimated using the Kimura80 approach and is denoted by *d* [30]. If we perform a regression analysis of the number of nucleotide differences (divergence) as a function of collection date, there should be a relation of the following form: *d* = *h_i_* * *μ* + *β*. This linear relationship between the variables indicates that the divergence between sample *i* and the bat coronavirus increases linearly with time. The regression intercept *β* indicates the divergence of the first sampled SARS-CoV-2 sequence in Wuhan from the bat CoV. To estimate the parameter *μ*, we calculated the slope of the regression line. Furthermore, the point at which this line crosses the x-axis (the axis of sampling dates) denotes the time point *t*_bat/Cov2_ at which the divergence between bat and SARS-CoV-2 is 0, i.e., the time of the most recent common ancestor between bat CoV and SARS-CoV-2. 

### 2.2. Recombination Analysis of Nonhuman Betacoronaviruses That Have Contributed to the SARS-CoV-2 Evolution and between the Human SARS-CoV-2 Genomes

Recombination evaluation was performed with a blast-based analysis. Let *DS* (Dataset Sars) denote the human SARS-CoV-2 genomes fasta dataset as it was downloaded from the GISAID database. Furthermore, *DC* (dataset coronavirus) denotes the dataset that comprises 55 Coronaviridae sequences downloaded from NCBI and the pangolin (GISAID accession ID: EPI_ISL_402131) and bat (GISAID accession ID: EPI_ISL_410721) sequences from GISAID (thus, 57 sequences in total). Also, *DBP* (dataset pangolin/bat) is a dataset with only two sequences: the bat and the pangolin sequence from GISAID. First, using *DS*, we generated a new dataset called *DSS* (dataset SARS split), which comprises all the unique 300-mers from the whole *DS*. To detect potential contribution of bat/pangolin-recombinant sequences into human SARS-CoV-2 genomes, we blasted the *DSS* dataset against the *DBP* dataset (i.e., *DBP* is the blast database, and *DSS* is the query set). If a sequence *DSS_i_* (or an ancestral sequence of it) is the result of recombination between the bat coronavirus sequence and the pangolin coronavirus sequence, then the sequence is matched to both bats and pangolins in consecutive parts. Thus, if *DSS_i_* is a sequence of length *L* (here *L* = 300 bp), we look for blast results of the following form: *DSS_i_* [*a*, *b*] matches bat/pangolin and *DSS_i_* [*c*, *d*] matches pangolin/bat, where 1 ≤ *a* ≤ *b* ≤ *c* ≤ *d* ≤ *L*. We applied also the following restrictions: 1 ≤ *a* ≤ 10, *L*-10 ≤ *d* ≤ *L*, |*b* − *c*| ≤ 20. In other words, *a* should be at the beginning of the 300-mer, *d* should be at the end of the 300-mer, and the distance between the bat-like and the pangolin-like parts should be smaller than 20. Further details on the recombination detection process are provided in Supplementary Materials Text S1 and Figure S1. 

To detect other potential recombination events, we used as a blast database the DC dataset to search for recombinants from other hosts besides bat and pangolin. Furthermore, to detect recombination between human SARS-CoV-2 sequences or their unsampled ancestral CoVs sequences, we used the DS dataset as a blast database. The specific parameters for running blastn were the following: *-outfmt 6* (to present the results in a tabulated format), *--word_size 10* (to capture even small fragments), and *-evalue 0.1* (to capture small fragments). 

### 2.3. Linkage Disequilibrium (LD) in Human SARS-CoV-2 Genomes

An indirect approach to inferring recombination in a sequence is the decay of linkage disequilibrium (LD) as a function of distance. Given the demographic model, LD between two sites may decrease either due to recombination events or due to recurrent mutations, thus violating the infinite site model [31]. From these two causal reasons of LD decay, only recombination decreases LD as a function of the distance between two sites since the recombination rate is proportional to the distance of the two sites. We used plink to calculate *r*^2^ [32] as a measurement of the haplotypic LD between all possible pairs of polymorphisms in the SARS-CoV-2 genome. Then, we modeled LD as a linear function of distance (assuming that the recombination rate is constant along the genome) as follows:

*r*^2^ = *ax* + *b* (1)

where *x* is the distance between two sites, and *a* and *b* are the slope and intercept, respectively.

If *a* is negative, then it supports the presence of recombination on the SARS-CoV-2 genome. On the other hand, if *a* is not statistically different from 0, then recombination may not be a reason for LD patterns along the genome.

### 2.4. Selective Sweeps and Common Outliers

To estimate potential targets of selective sweeps, we deployed the software tools SweeD [33] and RAiSD [34]. A selective sweep analysis detects characteristic patterns of polymorphisms attributed to the action of recent and strong positive selection from a new allele. The key mechanism that generates such polymorphic patterns is the fast increase in the beneficial mutation and the recombination rate [35]. Currently, the whole population of human SARS-CoV-2 is rapidly expanding. Furthermore, even though recombination may be present in viruses, our analyses did not find evidence of recombination among human SARS-CoV-2 sequences. This outcome does not suggest that recombination is absent but rather that diversity within the human SARS-CoV-2 is not large enough to allow for the detection of recombination events. Thus, a selective sweep analysis may still be meaningful for SARS-CoV-2. 

We performed a selective sweep analysis for each separate population (Asia, Europe, North America, South America, Oceania, and Africa) and for the total worldwide sample. SweeD was executed using its default parameters and a grid size of 5000 positions along the genome (-*grid* parameter), which led to the calculation of a composite likelihood ratio (CLR) score every 6 nucleotides, on average. Due to the tool’s high computational complexity for computing the site frequency spectrum, which can lead to prohibitively long execution times when the sample size increases, we used a smaller sample size for the worldwide analysis. The SweeD-based analysis of the employed dataset, which comprised 1601 genomes, required 112 CPU hours. Initial analyses with smaller sample sizes (performed as data and available online for a period of three weeks) revealed that SweeD results remain largely identical when the sample size exceeds 1000 sequences.

Unlike SweeD, which relies on maximum-likelihood estimation at predefined positions along the data, RAiSD computes the *μ*-statistic using a polymorphism-driven sliding-window algorithm. RAiSD was executed using a sliding-window size of 8 polymorphisms (-*w* parameter) and a step of 1 polymorphism (default). We also increased the slack for the SFS edges to 2 (*-c* parameter), which directed the tool to also consider doubletons and polymorphisms that belong to the *S*-2 class in the calculation of the *μ*_SFS_ factor, where *S* is the sample size. The default value for the SFS edge slack is 1, considering only singletons and polymorphisms in the S-1 class.

To conduct a common-outlier analysis based on the results obtained by SweeD and RAiSD, we implemented a series of extensions directly in the source code of RAiSD. More specifically, we introduced a new parameter (-*G*) that specifies the grid size similarly to SweeD, which directs RAiSD to report scores at the same positions as SweeD when the same grid size is given. Given the RAiSD polymorphism-driven approach, however, scores for the required positions are calculated using linear interpolation based on the initially evaluated positions. To detect common-outlier regions, we sorted the evaluated positions in each of the tool reports based on their associated scores and set the top 0.05 value as the cutoff threshold per method. This allowed us to identify all outlier positions per tool. Thereafter, we computed all pairwise base distances between SweeD and RAiSD outlier positions and reported candidate outlier position pairs if the evaluated positions were closer than 400 bases. The common-outlier analysis was integrated as a feature in RAiSD to facilitate reproduction of the results. The following example command line was used to detect common-outlier regions:

RAiSD -I inputFile -n runName -CO SweeDReport 1 2 -COT 0.05 -O -COD 400 -c 2 -w 8 -G 5000.

The -*CO* parameter provides a path to the SweeD report and the column indices for the positions and the scores. The *-COT* and *-COD* parameters specify the cutoff threshold for the outliers and the maximum distance between SweeD/RAiSD outlier positions, respectively.

### 2.5. Estimation of the Time of the Most Recent Common Ancestor

To estimate the time of the most recent common ancestor, we applied the following: Let *x*_0_ be the sequence of SARS-CoV-2 from the first reported patient in Wuhan. The sampling date was 24 December 2019. Any other sequence *y_i_* of sample *i* has a common ancestor with *x*_0_ at some time *t_i_* earlier than the date 24 December 2019. To assess *t_i_*, we need to consider the differences (diversity), *δ_i_*, between *x*_0_ and *y_i_*. The older it is, the more differences on average will exist between *x*_0_ and *y_i_*. In general, the expected number of differences, *δ_i_*, between *x*_0_ and *y_i_* is *δ_i_* = (*D_i_* + 2*t_i_*)*μl*, where *μ* is the mutation rate per base per day, *l* is the length of the genome (here, we used the value 30,000 bp), and *D_i_* is the number of days between the sampling date of *x*_0_ and *y_i_*. Substituting *δ_i_* with the observed number of pairwise differences between *x*_0_ and *y_i_*, *μ* = 1.87 × 10^−6^ (see the subsection “Estimation of mutation rate and divergence from bat” in the Results Section), we can estimate the parameter *t_i_* for sample *i*. Finally, we estimated the expected value of *t_i_* and the variance *V_t_* of *t_i_* as the sample average of the *t_i_* values and the sample variance, respectively. The reason behind using the first reported patient in Wuhan is that the common ancestor between this sequence and any other sequence should be either on 24 December 2019 or earlier than this date. On the contrary, it is possible that any two different sequences *y_i_* and *y_j_* (sampled later during the pandemic) have a common ancestor more recently than the date 24/12/2020; thus, it would not be possible to use them in order to estimate the time of the most recent common ancestor. 

### 2.6. Demographic Inference

The demographic analysis was performed in an approximate Bayesian computation (ABC) framework. ABC is a Bayesian approach that bypasses the exact likelihood computation by using stochastic simulations and summary statistics. [24]. In our analysis, we modeled the demography of the SARS-CoV-2 dataset that infected Asian, European, and Northern American cohorts. An important approximation in an ABC analysis is the replacement of the full data by a set of summary statistics. These are numerical values calculated from the data such that they represent the maximum amount of information in the simplest possible form [36]. The summary statistics were calculated from the multiple sequence alignment (MSA) of each population’s sequences by msABC [37] and include (1) estimates of genetic diversity—the Watterson’s estimator *θ_w_* [38] and the mean pairwise differences of sequences *θ_π_* [39]—(2) a summary of the site frequency spectrum in the form of Tajima’s D [40], (3) the average pairwise correlation coefficient *ZnS* [41] as a measure of linkage disequilibrium, (4) two haplotype-based statistics [42]—the number of haplotypes calculated by the Depaulis and Veuille K (DVK) and the haplotype diversity measured by the Depaulis and Veuille H (DVH)—and (5) the Thomson estimator of the time of the most recent common ancestor (TMRCA) [43] and its variance [44].

In order to estimate the parameters of a demographic scenario involving an exponential growth in the population size, we created simulations of neutral polymorphism data. Inferring the demographic history of one population at a time, we generated sets of 500,000 coalescent simulations using msABC [37]. In that framework, every evolutionary scenario was defined by a set of parameters and every parameter was characterized by a prior distribution (Table 1). Given the current effective size of a haploid population N_0_, the expansion model was characterized by two parameters: the scaled population mutation rate *θ* and the rate of exponential expansion *α*. Therefore, the population size is given by the following:

N(t) = N_0_ e^−*α*t^ (2)

where time t is measured backwards in time in units of 2N_0_ generations and *θ* is defined as 2N_0_*μ* for haploid organisms, where *μ* is the mutation rate per generation [45]. Since there was no clear evidence that recombination took place within SARS-CoV-2 genomes in humans, the population recombination rate *ρ* of all the simulations was set to zero. For each population, the number of individuals sampled per simulation was equal to the number of sequences in the multiple sequence alignment.

The ABC parameter inference was implemented using the R package abc [46]. The inference procedure consists in retaining simulations for which the Euclidean distance between the set of simulated summary statistics and the observed set is sufficiently small. The percentage of accepted simulations is determined by the tolerance value, *τ* = 0.005. Ιn order to correct for the discrepancy between the simulated and the observed statistics due to the nonzero *τ*, the posterior probability of the parameters was approximated by applying regression adjustment techniques to the retained simulations. More specifically, we performed a local linear weighted regression correction to estimate the posterior parameter probabilities of the American and Asian SARS-CoV-2 populations. In this approach, the simulated parameters were assigned weights according to how well the corresponding summary statistics adhered to the observed ones and then a linear regression adjustment was performed on the accepted, weighted parameters [24]. The parameters were “logit” transformed prior to estimation, using as boundaries the prior distributions’ minimum and maximum values that were used during the simulations for each parameter. After the regression estimation, the parameters were back-transformed to their original scale. A nonlinear heteroscedastic regression model using a feed-forward neural network model was utilized in the demographic analysis of the European population [47].

## 3. Results

We used publicly available sequence data from the GISAID database, downloaded on 2 April 2020, to infer the evolution of human SARS-CoV-2 strains circulated during the first wave of the pandemic. All analyses except selective-sweep localization were performed on this dataset. The initial dataset comprised 3230 full genome assembled sequences. From those, we kept only the high-coverage sequences (as defined by GISAID, these sequences contain less than 1% ambiguous states (N’s) and less than 0.05% unique amino acid mutations, i.e., not seen in other sequences in the database and no insertion-deletions unless verified by submitters). In total, 2305 sequences passed this filter. We applied additional filters to keep only high-quality sequences: First, we trimmed the ambiguous N’s from both the beginning and the end of the genomes. Then, after trimming, we kept only sequences with less than 10 ambiguous states (N’s). The final dataset consists of 1895 human SARS-CoV-2 genomes and two outgroup sequences: the bat CoV (hCoV-19/bat/Yunnan/RaTG13/2013; accession ID EPI_ISL_402131) and pangolin CoV genomes (hCoV-19/pangolin/Guangdong/1/2019; accession ID: EPI_ISL_410721). The dataset was aligned using the MAFFT software v.7.205 [48]. 

### 3.1. Estimation of Mutation Rate and Divergence from Bat CoV

**Figure 1:**
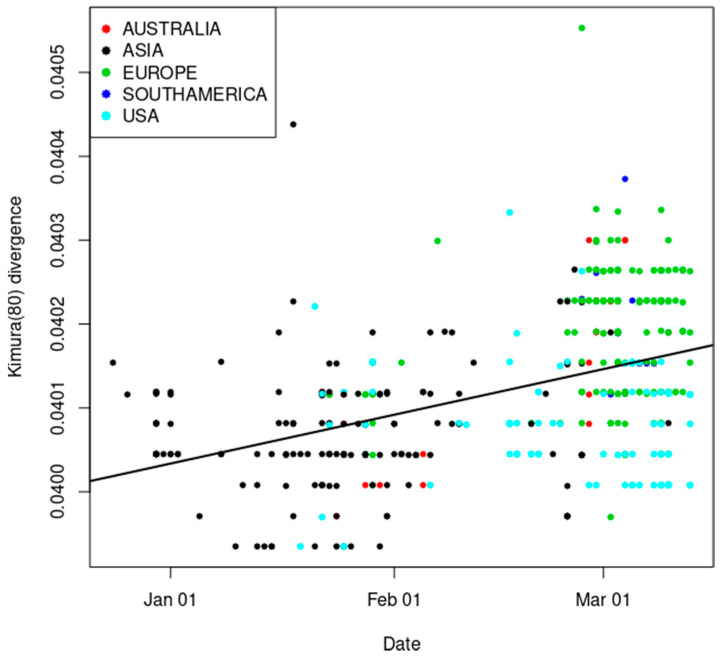
Regression analysis of the divergence from the bat coronavirus (CoV) genome as a function of sampling date: the slope of the regression line (1.87 × 10^−6^) reflects the increase in divergence per site and per day.

We modeled the divergence from the bat CoV genome as a function of the sequence sampling date. Divergence was estimated using the Kimura80 approach [30,49]. Since the sampling date was provided by the GISAID database, we were able to perform a regression analysis of the divergence per site as a function of the sampling date. The slope of the line represents the increase in divergence per site and per day; thus, it may be used as a proxy for the mutation rate per site and per day. The estimated mutation rate is 1.87 × 10^−6^ per nucleotide substitution per site per day (Figure 1). The divergence from the bat CoV genome is estimated to 0.04 substitutions per site using the Kimura (1980) calculation. Assuming that the mutation rate is 1.87 × 10^−6^, the total time since that separates SARS-CoV-2 from the bat CoV genome is 0.04/1.87 × 10^−6^ = 58.6 years. Thus, assuming an equal mutation rate between the bat CoV lineage and the human CoV genome, the time since the common ancestor between the bat CoV and the SARS-CoV-2 is about 58.6/2 = 29.3 years. 

### 3.2. Recombination Events

#### 3.2.1. Host Analysis

**Figure 2:**
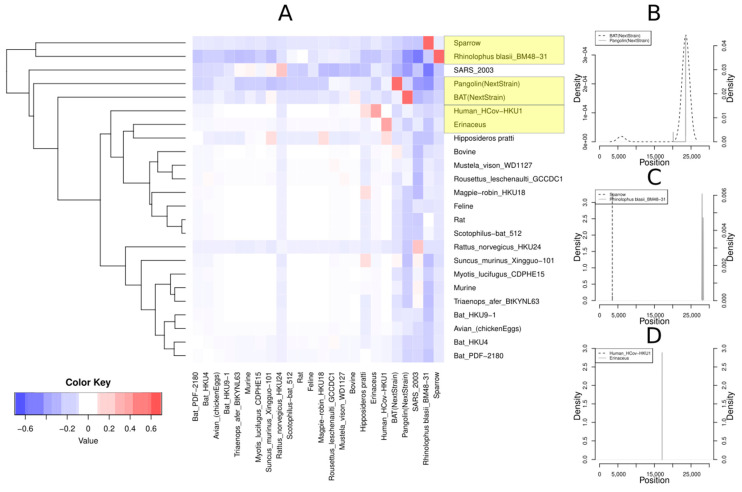
(**A**) Hosts of the Coronaviridae virus family in which plausible recombination events may have taken place: red-colored squares denote positive correlation coefficient values and, thus, the cooccurrence of blast results from different hosts for the same query sequence, suggesting potential recombination events. The blue and white squares denote pairs with negative or close to zero correlation coefficient. The yellow boxes denote the host pairs with the highest *r* values: pangolin (GISAID accession ID EPI_ISL_402131) and bat (GISAID accession ID: EPI_ISL_410721), sparrow (RefSeq ID: NC_016992.1), and *Rhinolophus blasii* (RefSeq ID: NC_014470.1), and the human CoV sequence (RefSeq ID: NC_006577.2) with hedgehog (RefSeq ID: NC_006577.2). Localization of recombination events in the (**B**) bat/pangolin pair, (**C**) sparrow/Rhinolophus pair, and (**D**) hedgehog/Human_CoV-HKU1.

We performed a blast-based analysis using as a (locally constructed) database the 55 fully sequenced Coronaviridae genomes downloaded from NCBI [50] together with the bat CoV and pangolin CoV genomes from the GISAID database. The query comprises all the unique 300-mers from the 1895 human SARS-CoV-2 genomes. In total, there are 452,212 unique 300-mers. The word size parameter of blast was set to 10 and the e-value was set to 0.1 to capture even small parts of the sequence that may resemble parts from the Coronaviridae family of different hosts. We checked whether the consecutive regions in a query sequence could match different entries in the local blast database, that is, Coronaviridae sequences from different hosts. Then, we estimated all correlation coefficients for all host pairs to evaluate their tendency to cooccur as a match in the same query sequence (Figure 2). For example, pangolin and bat cooccur in 395 out of 5568 total cases, resulting in an *r* value of 0.4. Such a high cooccurrence may suggest that an ancestral sequence of a query 300-mer could have experienced a recombination event in these two hosts. We found plausible past recombination events between CoVs from pangolin (GISAID accession ID: EPI_ISL_402131) and bat (GISAID accession ID: EPI_ISL_410721), sparrow (RefSeq ID: NC_016992.1) and *Rhinolophus blasii* (RefSeq ID: NC_014470.1), and the Human_CoV-HKU1 sequence (RefSeq ID: NC_006577.2) with hedgehog *(Erinaceus)* (RefSeq ID: NC_006577.2). 

#### 3.2.2. Localization of Recombination Events

For the three pairs of hosts depicted in Figure 2, we localized the genomic regions that may be involved in recombination events. Thus, for all concordant pairs, that is, pairs corresponding to consecutive matches between a query 300-mer and two different database entries, we used the genomic coordinates of the non-SARS-CoV-2 host. The density plots in Figure 2B–D illustrate the distribution of the location in the genome of the host. For the pangolin/bat pair (Figure 2B), the location of both pangolin and bat fragments are around the position 23,583, suggesting a plausible recombination event in the highly divergent Spike protein S1 coding region. On the other hand, the sparrow/Rhinolophus pair shows different localization from the sparrow and Rhinolophus fragments. The sparrow fragments are located around the location 3484, whereas the Rhinolophus fragments are located around the position 28,034. Finally, for the hedgehog/Human_HCoV-HKU1 pair, both locations are around the position 17,000 (17,062 and 17,088, respectively), within the ORF1b polyprotein and the helicase/nsp13 coding region. 

### 3.3. Detection of Recombination Events amongst Human SARS-CoV-2 Genomes

Using a similar approach as in “Host analysis”, we assessed whether recombination events took place between the human SARS-CoV-2 genomes. No recombination event was detected using the human SARS-CoV-2 genome sequences from the GISAID database until the date 2 April 2020.

### 3.4. Linkage Disequilibrium (LD) Analysis

An indirect approach to inferring recombination in a sequence is the decay of LD as a function of distance. We estimated LD between all sites using the software *plink* [51], and we modeled haplotypic LD as a function of distance between two genomic sites using a simple linear model:

LD = *ax* + *b* (3)

where *x* is the distance. The analysis was performed on the SARS-CoV-2 genomes for each population separately (Europe, Africa, North America, South America, Oceania, and Asia). The results for parameters *a* and *b* are presented in Table S1. These results suggest that, for the European, Asian, and total populations, LD decreases as a function of distance. Thus, recombination may have occurred in SARS-CoV-2 genomes. These findings are not contradictory to the previous subsection “Detection of recombination events between the SARS-CoV-2 genomes”. In the previous section, our goal was to detect the recombination breakpoints and the recombinant sequences (or the descendants of the recombinant sequences). However, if the sequences are not divergent enough, such an approach will not reveal the breakpoints. On the contrary, the LD decay approach is more sensitive to showing that recombination may have occurred (however, it can reveal neither the recombination breakpoints nor the recombinant sequences). Table S1 shows that, in the North America, South America, Oceania, and Africa populations, the value of the slope *a* is not statistically different from 0, i.e., there is no correlation between the genomic distance and the LD. This is probably due to the fact that the number of recombination events that take place in a population is a function of both the recombination rate and the effective population size (*ρ* = 4Ν_e_*r*, where N_e_ is the effective population size and r is the recombination rate). Since the exponential growth phase started later in these continents compared to Asia and Europe, it is possible that the effective population size of the virus was not large enough for recombination to be detected in the North America, South America, Oceania, and Africa populations. 

### 3.5. Selective Sweeps

Selective sweep analyses were conducted on the dataset downloaded on 31 March 2020 from an early stage of the epidemic. We used SweeD [33] and RAiSD [34] to identify potential targets of selective sweeps. SweeD implements a CLR test based on the site-frequency spectrum, while RAiSD evaluates the *μ*-statistic that relies on multiple sweep signatures. Figure S10 illustrates the scores of both tools along the genome when analyzing 1601 SARS-CoV-2 sequences (version 31 March 2020) in the total population and per continent for Europe, Asia, and North America. The figure also shows the top 5% common outliers. Given the accuracy granularity of existing sweep detection methods, including the employed ones in this study, we refrained from identifying specific genomic positions at site granularity.

Table 2 provides the distinct region pairs that were identified as potential sweep targets. For the world sample, the first two region pairs are located within the nonstructural protein 3 (nsp3). The third region pair belongs to Spike protein S1, which attaches the virion to the cell membrane by interacting with a host receptor, thereby initiating the infection. The highly divergent Spike protein S1 of coronaviruses facilitates viral attachment, fusion, and entry and thus serves as a target for antibody and vaccine development [52]. The identified outlier region 23,043–23,073 falls into the receptor-binding domain (RBD), which has been reported to be optimized as a result of natural selection for binding to human angiotensin-converting enzyme 2 (ACE2) receptors and consists of a putative target for designing future therapeutic or preventive strategies [6]. The fourth region pair is in the spike protein S2. 

### 3.6. Summary Statistics along the SARS-CoV-2 Genome

**Figure 3:**
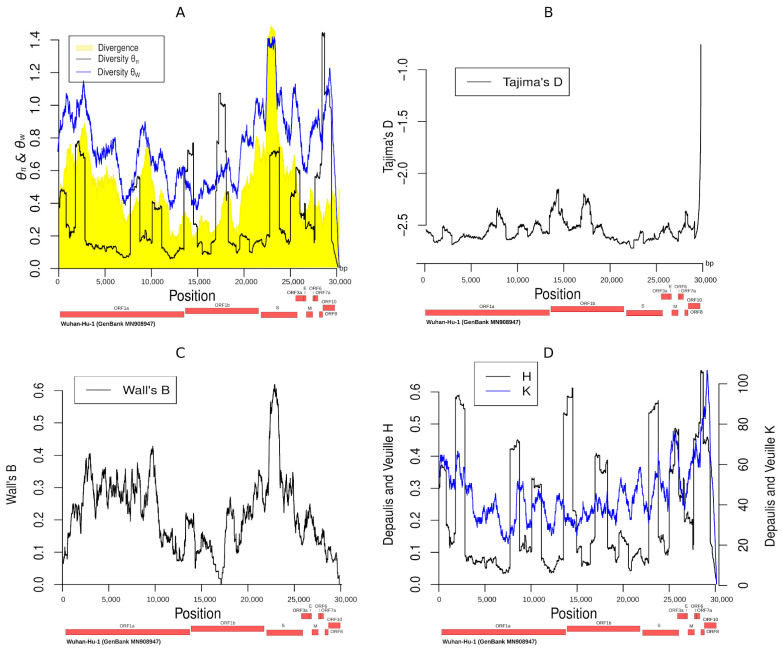
Distribution along the genome of the summary statistics *θ_π_, θ_W_*, divergence (**A**), Tajima’s D (**B**), Wall’s B (**C**), and Depaulis and Veuille H and K (**D**). Wall’s Q is similar to Wall’s B and is not shown in the figure.

We estimated the values of several summary statistics (Figure 3) commonly used in population genetics studies along the genome of SARS-CoV-2: (i) Watterson’s estimate [38] of *θ* value (the population mutation rate); (ii) *θ_π_* [39], i.e., the average number of pairwise differences in the sampled sequences, which is another estimate of *θ*; (iii) Tajima’s D [40], a commonly used neutrality test that receives negative values typically in cases of population expansion and/or recent strong positive selection; (iv) Wall’s B and Wall’s Q [53], which are related to the recombination rate; (v) the divergence to the bat sequence using a random SARS-CoV-2 genome and the average divergence from the bat Coronavirus sequence using all SARS-CoV-2 genomes; and (vi) the Depaulis and Veuille K and H statistics [42], which denote the number of haplotypes and the haplotype heterozygosity, respectively. The region around position 23,000 (Spike protein S1/RBD) is characterized by large divergence, low Tajima’s D, and high values of Wall’s B and Q. The divergence values (high) and the Tajima’s D values (low) suggest that it may be a target of recent positive selection. The value of *θ_W_* is high, which is due to the excessive number of singletons in the region since *θ_π_* does not receive very high values. This result is consistent with positive selection, even though we cannot exclude artifacts from sequencing errors or more complex evolutionary phenomena (e.g., recombination with another host). Another probable explanation is that selective sweep is ongoing. Thus, several low-frequency polymorphisms remain with decreasing Tajima’s D and high values for *θ_W_*.

### 3.7. Estimation of the Time of the Most Recent Common Ancestor

We used all samples to estimate the mean and the variance of the parameter *t* (Figure S2). The mean parameter value estimation was *t_est_* = 47.0, and the variance was *V_est_ =* 504.45. Figure S2B depicts the histogram, density, and the mean value for the parameter *t*, the time of the most recent common ancestor measured in days before the first Wuhan sample (24 December 2019). The estimated value of *t* suggests that the most recent common ancestor of SARS-CoV-2 genomes is older (47 days) than the first reported case, dating the TMRCA to November 7th, 2019. Even though it is reasonable that the first case is dated earlier than the first reported case, here, we cannot know whether the TMRCA refers to a human sample or another host (e.g., bat).

### 3.8. Demographic Inference

Exploring the demographic dynamics of SARS-CoV-2 populations is important in understanding the magnitude of the spread of the ongoing COVID-19 pandemic. Here, we focused on the SARS-CoV-2 sequences of Asian, European, and Northern American human cohorts, and we employed an approximate Bayesian computation (ABC) approach to model the exponential growth of the three SARS-CoV-2 populations. For each population, the parameter inference procedure involves the estimation of the posterior distributions of two parameters: the scaled population mutation rate *θ* and the rate of exponential expansion *α*. In this model of population growth, the population size is given by N(t) = N_0_ e^−a**t**^, where time t is measured backwards in time in units of 2N_0_ generations and *θ* is defined as 2N_0_*μ* for haploid organisms, where *μ* is the mutation rate per generation and N_0_ the present-day effective population size [45].

For each population under study, ABC employs stochastic coalescent simulations to estimate the properties of the parameters’ posterior distributions. The parameter inference procedure consists in retaining only the best *τ* of the simulations, i.e., *τ* of the simulated datasets, which are closest to the observation (Figures S3–S5). Visualization of the percentage of accepted simulations that was determined with a tolerance value of 0.005 (Figures S6–S8) in a contour plot demonstrates how these simulations explore the space around the observed data. The summary statistics form an 8-dimensional space. For some statistics (i.e., dimensions), the contour lines diverge from the observed value because the algorithm only retains the simulations that minimize the 8-dimensional Euclidean distance to the observed vector. Thus, even if the distance is minimized over all dimensions, this may not be true for each dimension separately. For example, the posterior distributions of Tajima’s D and DVK of the European population, in particular, show that the accepted values (within the tolerance *τ*) are quite distant from the observed value. Specifically, Tajma’s D and DVK are affected by low-frequency-derived variants, especially singletons. Tajima’s D becomes negative and DVK assumes large values in the presence of singletons since the number of haplotypes increases. In the SARS-CoV-2 dataset, a large proportion of polymorphisms (>70%) are singletons, which results in extreme negative values of Tajima’s D. Such a proportion of singletons is difficult to explain with a simple demographic model of population expansion alone. It is possible that other factors (e.g., sequencing errors and more complex demographic models) have contributed to such an observation.

**Figure 4:**
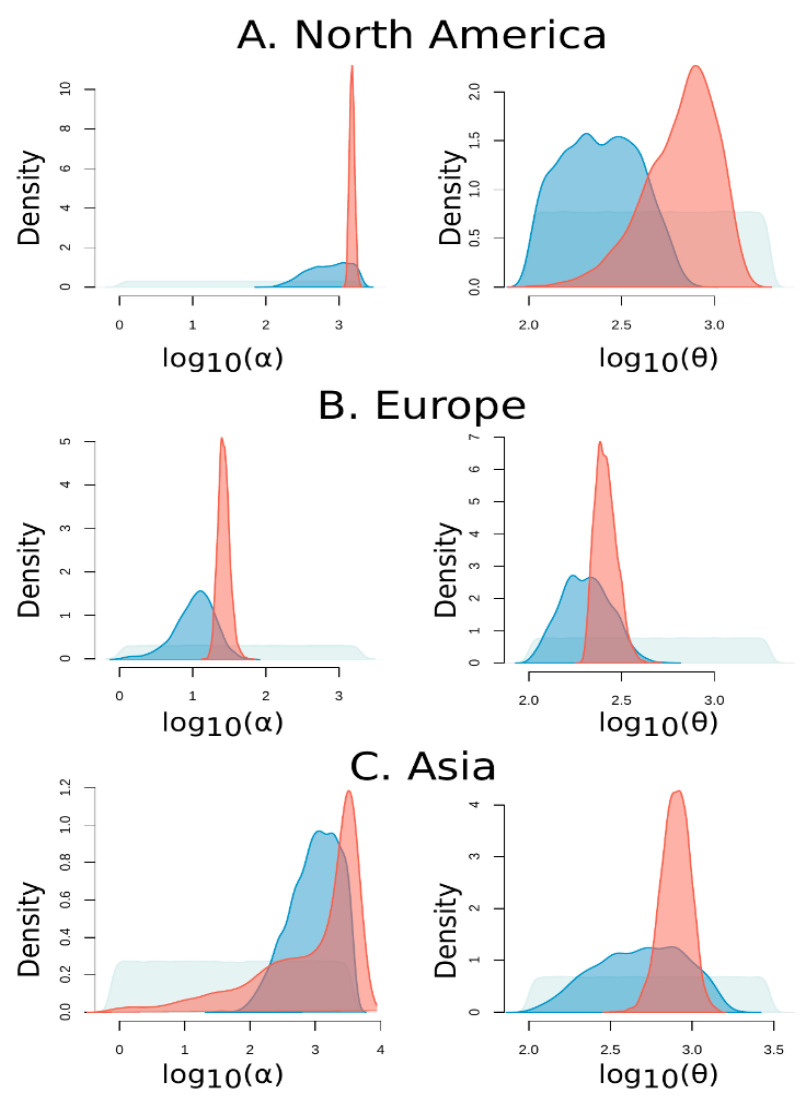
Probability estimations of (**A**) North America’s, (**B**) Europe’s, and (**C**) Asia’s SARS-CoV-2 population expansion parameters. Grey curve: prior parameter distribution; blue curve: density distribution of the approximate Bayesian computation (ABC)-unadjusted simulation values that were accepted with a tolerance of 0.005; and red curve: posterior distribution approximated by applying regression correction to the retained simulations.

An improvement to the simple rejection ABC algorithm was employed to correct for the discrepancy between the simulated and the observed statistics. The posterior probability of the parameters was approximated by applying regression adjustment techniques to the retained simulations. More specifically, we performed a local linear-weighted regression correction to estimate the posterior parameter probabilities of the American and the Asian SARS-CoV-2 population, while a nonlinear regression model using feed-forward neural networks was utilized in the demographic analysis of the European population. This correction influenced our estimates, reducing the variance of the posterior estimates (Figure 4). The inferred mean, median, and mode values as well as quantile values are shown in Table 3. Both *α* and *θ* values are higher in the Asian and North American populations than the European population. This suggests that population growth was faster in Asia and North America compared to the European population. Also, since *θ* = 2N_0_*μ* (N_0_ is the present day population size for each population) is higher for the Asian and North American populations, it suggests that Ν_0_ is higher in Asia and North America than Europe. We note that N_0_ is different from the effective population size: the effective population size N_e_ represents a long-term ideal population size while N_0_ refers only to the present. Furthermore, we note that, a posteriori, the two parameters *α* and *θ* are correlated (Figure S9). Thus, the likelihood surface may have multiple local optima and the inference process may result in different optima (and, therefore, parameter estimates) in different populations. 

## 4. Discussion

Population genetics approaches provide valuable insights into the evolutionary dynamics of the first wave of the COVID-19 pandemic. The mutation rate of the SARS-CoV-2 genome was inferred to be 1.87 × 10^−6^ nucleotide substitutions per site per day. This estimation is in line with other studies [54,55] as well as with what has been previously reported for SARS-CoV-1 (0.80 to 2.38 × 10^−3^ per site and per year, i.e., 2.24–6.68 × 10^−6^ per site and per day) and for other CoVs [56,57,58]. We further estimated that the TMRCA of the SARS-CoV-2 genomic sequences dates back to November 7th, 2019. Our TMRCA calculation comes in close proximity to recently published results [59], employed a molecular clock analysis, and assessed the TMRCA of the human outbreak to 25 November 2019 (95% highest posterior density (HPD) region: 28 September 2019; 21 December 2019). More recently, Liu and colleagues [55] presented a robust estimator of the mutation rate and the TMRCA that bypasses the effect of certain biases, such as sampling biases and existence of infection clusters during the epidemic spread. We reckon that our methodology also provides a similar estimate of those parameters, as the inferred values are similar to the results of [55]. Overall, those findings slightly differ with results from the application of Bayesian Markov Chain Monte Carlo (MCMC) approaches on data from the early outbreak [60] that propose a higher mutation rate (mean value of 6.14 × 10^−6^ substitutions per site per day), resulting in a more recent estimate of the TMRCA, on 11 December 2019. 

In terms of recombination events, our analysis revealed a plausible past recombination between animal CoVs from pangolin and bat, which has been also confirmed by similar analyses (i.e., Simplot and Recombination Identification Program (RIP) tool for recombination detection) [17,22,23]. Interestingly, we uncovered possible recombination events between sparrow and *Rhinolophus blasii* (bat), and the hCoV-HKU1 genome with hedgehog (*Erinaceus*), shedding light on another possible scenario about the origins of SARS-CoV-2. Localization of the pangolin and bat fragments were found around the position 23,583 inside the Spike protein S1 coding region, not surprisingly since the Spike region in Coronaviruses is known to be prone to recombination events, often including recombination hotspots [61]. Moreover, recently, Makarenkov et al., using a gene-by-gene phylogenetic-based approach, reported a detailed list of recombination events in the 11 ORFs of the SARS-CoV-2 genome. Similar to our analysis, they detected a recombination event between the RaTG13 and Pangolin CoVs in the Spike protein S1 [12]. In addition, they found evidence for between-species recombination in other genomic regions (e.g., the ORF1ab, S, ORF3a, ORF7a, ORF8, and N). A plausible explanation is that a phylogenetic-based analysis is more sensitive than a blast-based analysis. Similarly, Boni et al. [13] reported that SARS-CoV-2 shows frequent recombination in its evolutionary history, and they underlined its importance for the generation of the novel SARS-CoV-2 genomes. Additionally, they reported that such recombination events are old and that there is no evidence for very recent recombination events. However, in contrast to our and Makarenkov et al.’s analyses, their results indicate a single ancestral lineage circulating in horseshoe bats undergoing a complex recombination history. Interestingly, the sparrow/Rhinolophus pairs are localized differently from the sparrow and Rhinolophus fragments. The sparrow fragments are located around the location 3484, whereas the Rhinolophus fragments are located around the position 28,034. Thus, it is probable that an ectopic recombination event between ORF1a and ORF8 took place, since it is common for viruses to exchange genetic material in a nonreciprocal manner [11] even though we cannot exclude the possibility of a false positive result. The hedgehog/Human_HCoV-HKU1 pair locations, around the position 17,000, remained within the ORF1b non-structural polyprotein and the possible drug target helicase/nsp13 coding region [62]. 

In addition, we localized the action of positive selection by detecting selective sweeps in order to explore putative adaptive genetic regions of the SARS-CoV-2 genome. One of the positively selected sites is located inside the non-structural protein 3 (nsp3), which is a large multidomain and multifunctional protein that plays a key role in coronavirus replication. Previous studies reported that the nsp3 represents a preferential selection target in MERS-CoV and lineage C betaCoVs [63], and more recent results support our findings and indicated that this region is quickly evolving in coronaviruses [64,65]. The third region pair was found to show positive selection belonging to Spike protein S1, which attaches the virion to the cell membrane by interacting with a host receptor, thereby initiating the infection. The highly divergent Spike protein S1 of coronaviruses facilitates viral attachment, fusion, and entry and thus serves as a target for antibody and vaccine development [52]. The identified outlier region 23,043–23,073 falls into the receptor-binding domain (RBD) and has been reported to be optimized as a result of natural selection for binding to human angiotensin-converting enzyme 2 (ACE2) receptors and to consist of a putative target for designing future therapeutic or preventive strategies [6]. This finding is in line also with the Cagliani’s results as well as other data [66]. The fourth region pair selected is in the spike protein S2, which mediates the fusion of the virion and cellular membranes by acting as a class I viral-fusion protein found also to be putative selective in previous analyses [64,65]. Even though some studies support that the signature of positive selection is weak at the early stages of the epidemic [67], our selective sweep detection method using SweeD and RAiSD shows that analyses in early sequences are able to detect positive selection. Similar analyses with different methods show a low ability to detect diversifying selection [68]. Our study revealed that strong positive selection was detectable in the early stages of the pandemic. Notably, these regions are of particular interest as they are more likely to reflect a functional change and vaccine putative targets, which may help early detection.

Finally, exploring the demographic dynamics of SARS-CoV-2 populations is important in understanding the magnitude of the spread of the COVID-19 pandemic. Past changes in the size of a population typically leave a signature on the SNP patterns of a present-day alignment. Our preliminary analysis on population genetics statistics provided an informative summary of the genetic diversity in our samples. An excess of low-frequency variants results in notably lower values of the average pairwise differences between sequences (*θ_π_*) compared to the (normalized) number of SNPs (*θ_W_*). For this reason, Tajima’s D appears to be skewed towards negative values and suggests an expansion in population size. This observation is in good agreement with previous findings, where a negative Tajima’s D was estimated from SARS-CoV-2 genomes [55], indicating a population expansion [69]. We therefore employed an ABC approach to estimate the exponential growth rate of SARS-CoV-2 sequences originated from Asian, European, and Northern American human cohorts during the months of the first wave. 

## 5. Conclusions

To conclude, our study suggests that (i) plausible past recombination events between CoVs from pangolin and bat have contributed in the early evolution of SARS-CoV-2, as has been previously reported by several other studies, and additionally, we report evidence for additional recombination events that involve coronavirus genomes from other hosts, i.e., hedgehog and sparrow; (ii) the SARS-CoV-2 genomic mutation rate is relatively low and was inferred to be 1.87 × 10^−6^ nucleotide substitutions per site per day, which is in line with several other studies as well as with what has been previously reported for BetaCoVs; (iii) in the European, Asian, and North American regions, the SARS-CoV-2 populations have accumulated an exponential growth during the first wave of the pandemic, which can support the observed polymorphism patterns in human SARS-CoV-2 genomes; and (iv) two regions within the spike and the nsp3 genes show evidence for recent positive selection.

## Figures and Tables

**Table 1:** Prior distributions for the demographic parameters.

Parameter	Prior Distribution		
	Minimum	Maximum	Distribution
**Northern American and European population**			
*θ*	100	2000	Log Uniform
*α*	1	2000	Log Uniform
**Asian population**			
*θ*	100	3000	Log Uniform
*α*	1	5000	Log Uniform

**Table 2:** Common-outlier region pairs between SweeD and RAiSD based on a maximal distance of 0.4 kb.

Population (No. of Sequences)	SweeD Outlier Region	RAiSD Outlier Region	Description
**All** (1601)	5276–5463	5048–5679	Non-structural protein 3 (nsp3)
	7102–7470	7380–7842	Non-structural protein 3 (nsp3)
	23,043–23,073	23,440–23,469	Spike protein S1/RBD
	24,749–24,773	24,762–24,814	Spike protein S2
**Europe** (811)	5094–5352	4695–5089	Non-structural protein 3 (nsp3)
	21,520–21,550	21,157–21,339	Non-structural protein 16 (nsp16)
	22,463–22,967	22,687–22,852	Spike protein S1
**Asia** (385)	7356–7815	7578–8182	Non-structural protein 3 (nsp3)
	16,057–17,022	15,925–16,714	nsp12/nsp13
**North** **America** (600)	8187–8337	7969–8220	nsp3
	12,523	12,450–12,923	nsp8/nsp9
	13,566–13,691	13,192–13,297	nsp12/nsp10
	15,147–15,201	15,248–15,383	nsp12
	23,166	23,398–23,427	Spike protein S1

**Table 3:** Summary statistics of the posterior distributions that were estimated by ABC for each SARS-CoV-2 population.

	North America		Asia		Europe	
	*α*	*θ*	*α*	*θ*	*α*	*θ*
**Weighted Mean 2.5% Percentile**	1299.12	235.37	12.91	523.95	19.26	211.95
**Weighted Median**	1500.96	709.24	2310.46	794.61	26.21	255.9
**Weighted Mean**	1509.05	717.89	2508.8	807.84	26.85	260.3
**Weighted Mode**	1507.6	733.87	4869.54	738.66	24.81	241.78
**Weighted Mean 97.5% Percentile**	1766.13	1300.2	4999.94	1147.67	38.63	333.24

## Data Availability

All submitters of data may be contacted directly via www.gisaid.org (accessed on 5 February 2021).

## Supplementary Materials

The following are available online at https://www.mdpi.com/2075-1729/11/2/129/s1, Figure S1: Identifying recombination events in SARS-CoV-2 genome data: all unique 300-mers from human SARS-CoV-2 sequences (seq1, seq2, seq3, and seq4) were aligned as a blast query against all available CoV sequences (blast database) from other species (bottom part of the figure). Recombination is detected if, around a location (here, denoted as “recombination position”) on the reference genome (black horizontal line on the top), there are 300-mers for which the left part (blue color) matches species A CoV whereas the right part (red color) matches species B CoV, Figure S2: The parameter t denotes the time of the most recent common ancestor as estimated in units of days prior to 24 December 2019, the collection date of the first Wuhan sample that was reposited in GISAID., Figure S3: ABC parameter inference for the European population based on a set of 8 summary statistics. Orange line: Observed value of each summary statistic; grey curve: density curve of the summary statistics calculated from the whole simulation space; and blue curve: density estimation of the ABC-accepted summary statistics that were determined with a tolerance τ = 0.005, Figure S4: ABC parameter inference for the Asian population based on a set of 8 summary statistics. Orange line: observed value of each summary statistic; grey curve: density curve of the summary statistics calculated from the whole simulation space; and blue curve: density estimation of the ABC-accepted summary statistics that were determined with a tolerance τ = 0.005, Figure S5: ABC parameter inference for the Northern American population based on a set of 8 summary statistics. Orange line: observed value of each summary statistic; grey curve: density curve of the summary statistics calculated from the whole simulation space; and blue curve: density estimation of the ABC-accepted summary statistics that were determined with a tolerance τ = 0.005, Figure S6: The space of the ABC-accepted simulations of the European population: the contour lines on the scatterplot indicate the density estimation, Figure S7: The space of the ABC-accepted simulations of the Asian population: the contour lines on the scatterplot indicate the density estimation, Figure S8: The space of the ABC-accepted simulations of the Northern American population: the contour lines on the scatterplot indicate the density estimation, Figure S9: Correlation of Asia’s population α and θ parameters of the ABC-accepted simulations (unadjusted values): the Pearson correlation coefficient is 0.95, Figure S10: Common outliers between SweeD and RAiSD (top 0.05): (A) world sample (1895 genomes), (B) Europe (811 genomes), (C) Asia (385 genomes), and (D) North America (600 genomes); Table S1: Linkage disequilibrium linear model results: with bold face fonts, we denote the populations characterized by *p*-values < 0.01 for the a parameter (the slope of the linear model).
