# Supplementary material for: Population Genomics Insights into the First Wave of COVID-19
Source: Life (Basel). 2021 Feb 7;11(2):129. doi: 10.3390/life11020129 (PMC7914631; doi:10.3390/life11020129)
Supplement: Supplementary file 1 [file life-11-00129-s001.pdf]

life

MDPI

Supporting material

Supplementary text S1

The recombination detection process: As described in the main text (2.2 section in Materials and Methods), the blue region that matches species A CoV, should be in the beginning of the 300-mer, and the red part (matches species B CoV) should be at the end of the 300-mer. The choice of the 300 bps as the length of the *k*-mer was justified by experimentation. Short *k*-mers (e.g., 100-mers) were generating blast matches at various genome locations, whereas very large *k-mers* were often not able to match their entire length, either up to their beginning or end. However, it should be noted that the length of the recombinant sequence interval (300-mer) does not affect the results of the analysis considerably. If for example a small piece of Species B CoV has been recombined, then *seq1* and *seq4* (Figure S1) might not be able to match Species B CoV up to their end (since their "right" part might be too long), however other unique sequences (e.g. *seq2* and/or *seq3* that are characterized by a shorter "right" part) will give a match.

Table S1: Linkage Disequilibrium linear model results. With bold face fonts we denote the populations characterized by p-values < 0.01 for the *a* parameter (the slope of the linear model).

**Population**	***a* (p-value)**	***b* (p-value)**
All	-1.152e-07 (<2e-16)	3.944e-03 (<2e-16)
Europe	-1.905512e-07 (<2e-16)	6.344816e-03 (<2e-16)
North America	-3.172727e-08 (0.113)	5.861180e-03 (<2e-16)
South America	-2.704880e-07 (0.818)	1.967903e-01 (<2e-16)
Africa	3.3661e-06 (3.380589e-02)	1.4271e-01 (1.4423e-11)
Oceania	9.764460e-08 (0.648)	2.821038e-02 (<2e-16)
Asia	-1.494132e-06 (<2e-16)	3.608169e-02 (<2e-16)

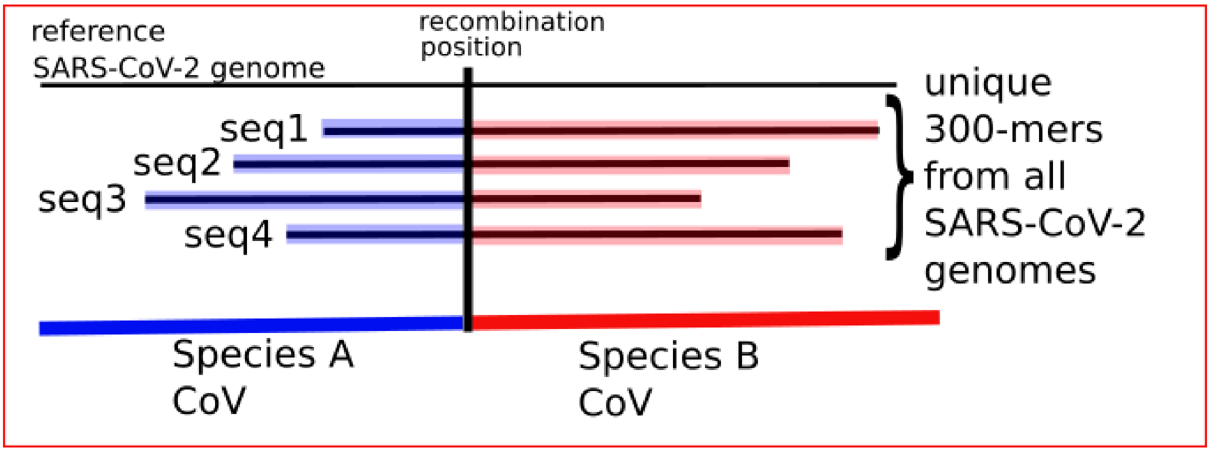

Figure S1. Identifying recombination events in SARS-CoV-2 genome data. All unique 300-mers from human SARS-CoV-2 sequences (seq1, seq2, seq3, seq4) were aligned, as a blast query, against all available CoV sequences (blast database) from other species (bottom part of the figure). Recombination is detected if around a location (here denoted as 'recombination position') on the reference genome (black horizontal line on the top), there are 300-mers for which the left part (blue color) matches species A CoV, whereas the right part (red color) matches species B CoV.

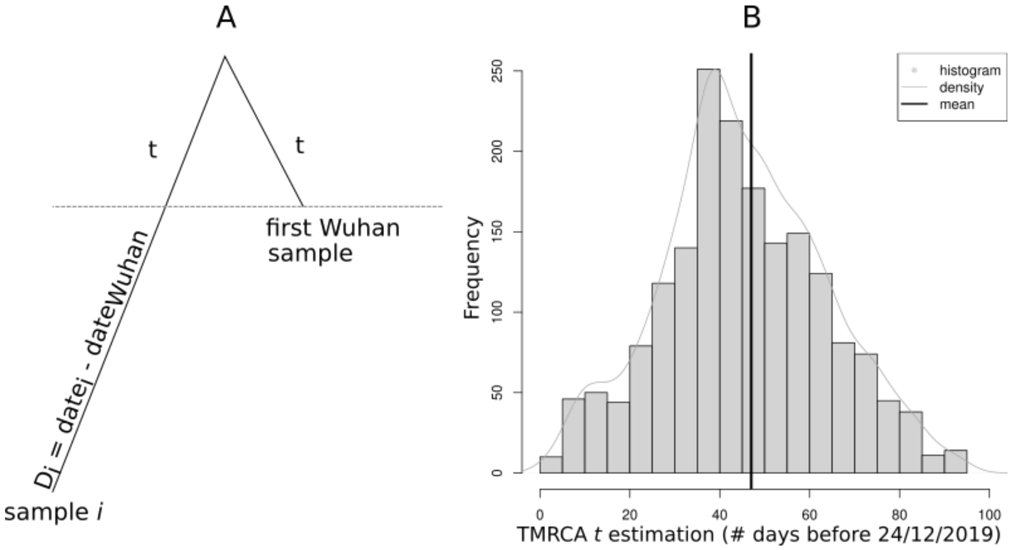

Figure S2: (A) The parameter *t* denotes the time of the most recent common ancestor as estimated in units of days prior to 24/12/2019, the collection date of the first Wuhan sample that was reposited in GISAID. (B) The mean parameter value estimation was t_{est} = 47.0, and the variance was 504.45. The histogram depicts the density and the mean value for the parameter *t*, the time of the most recent common ancestor measured in days before the first Wuhan sample (24/12/2019)

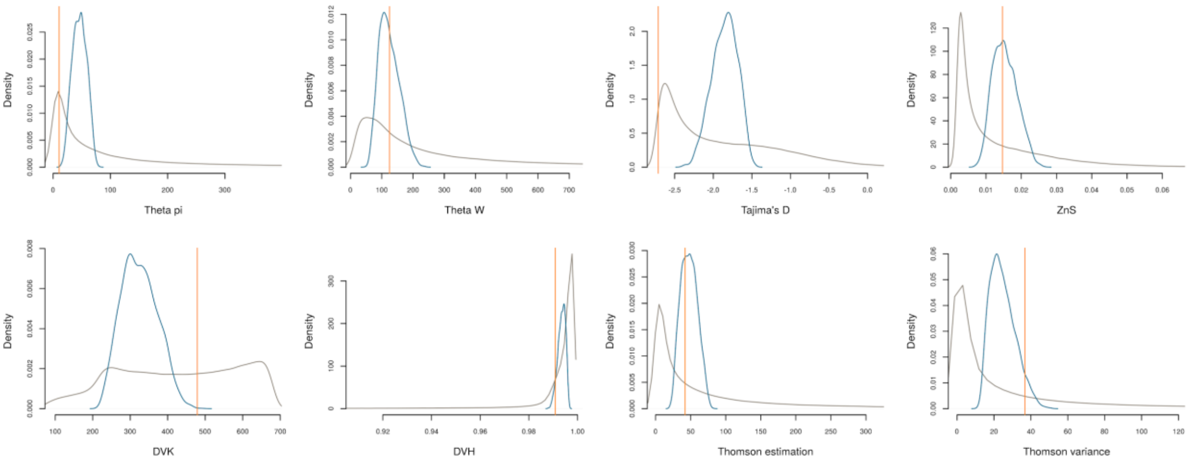

Figure S3: ABC parameter inference for the European population based on a set of 8 summary statistics. Orange line: Observed value of each summary statistic; Grey curve: density curve of the summary statistics calculated from the whole simulation space; Blue curve: density estimation of the ABC accepted summary statistics that were determined with a tolerance τ=0.005.

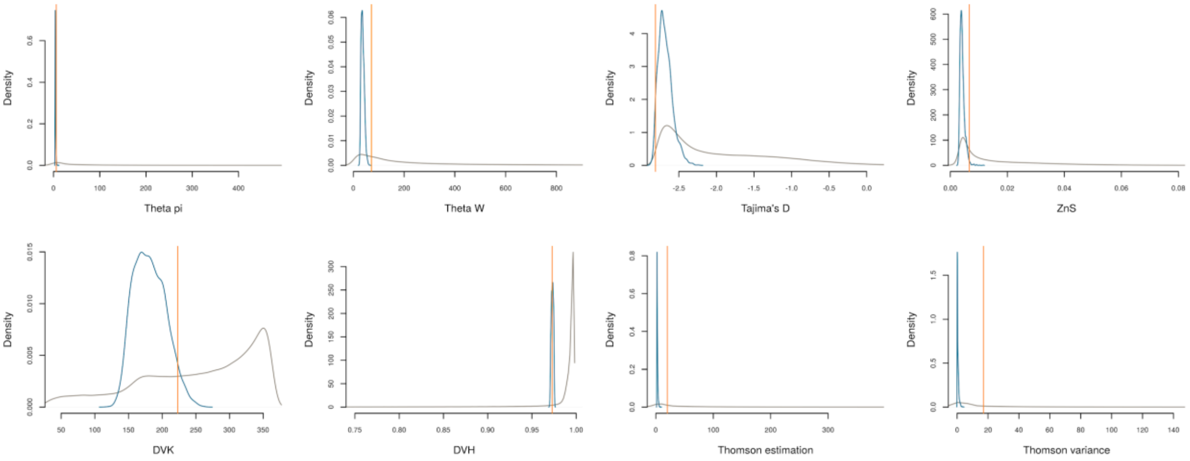

Figure S4: ABC parameter inference for the Asian population based on a set of 8 summary statistics. Orange line: Observed value of each summary statistic; Grey curve: density curve of the summary statistics calculated from the whole simulation space; Blue curve: density estimation of the ABC accepted summary statistics that were determined with a tolerance τ=0.005.

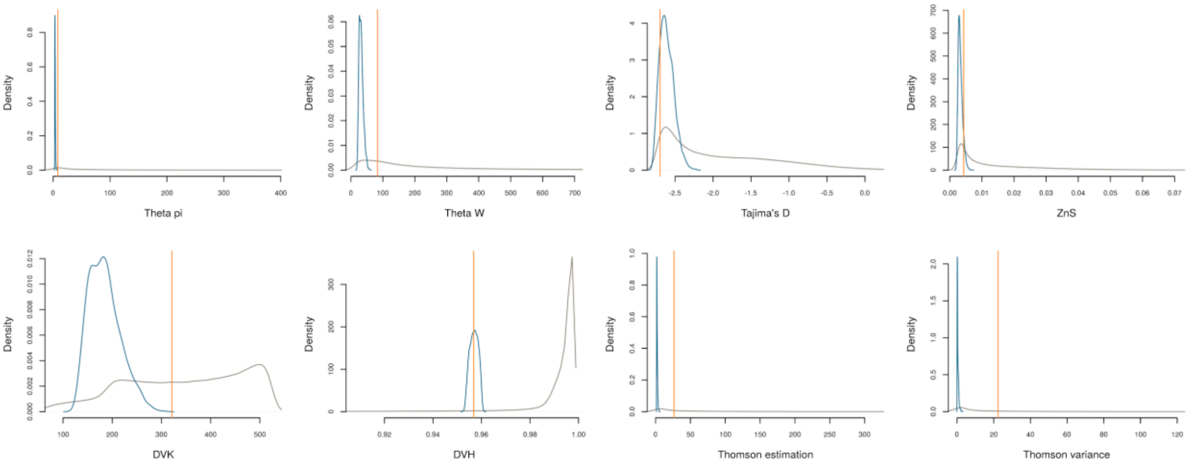

Figure S5: ABC parameter inference for the Northern American population based on a set of 8 summary statistics. Orange line: Observed value of each summary statistic; Grey curve: density curve of the summary statistics calculated from the whole simulation space; Blue curve: density estimation of the ABC accepted summary statistics that were determined with a tolerance τ=0.005.

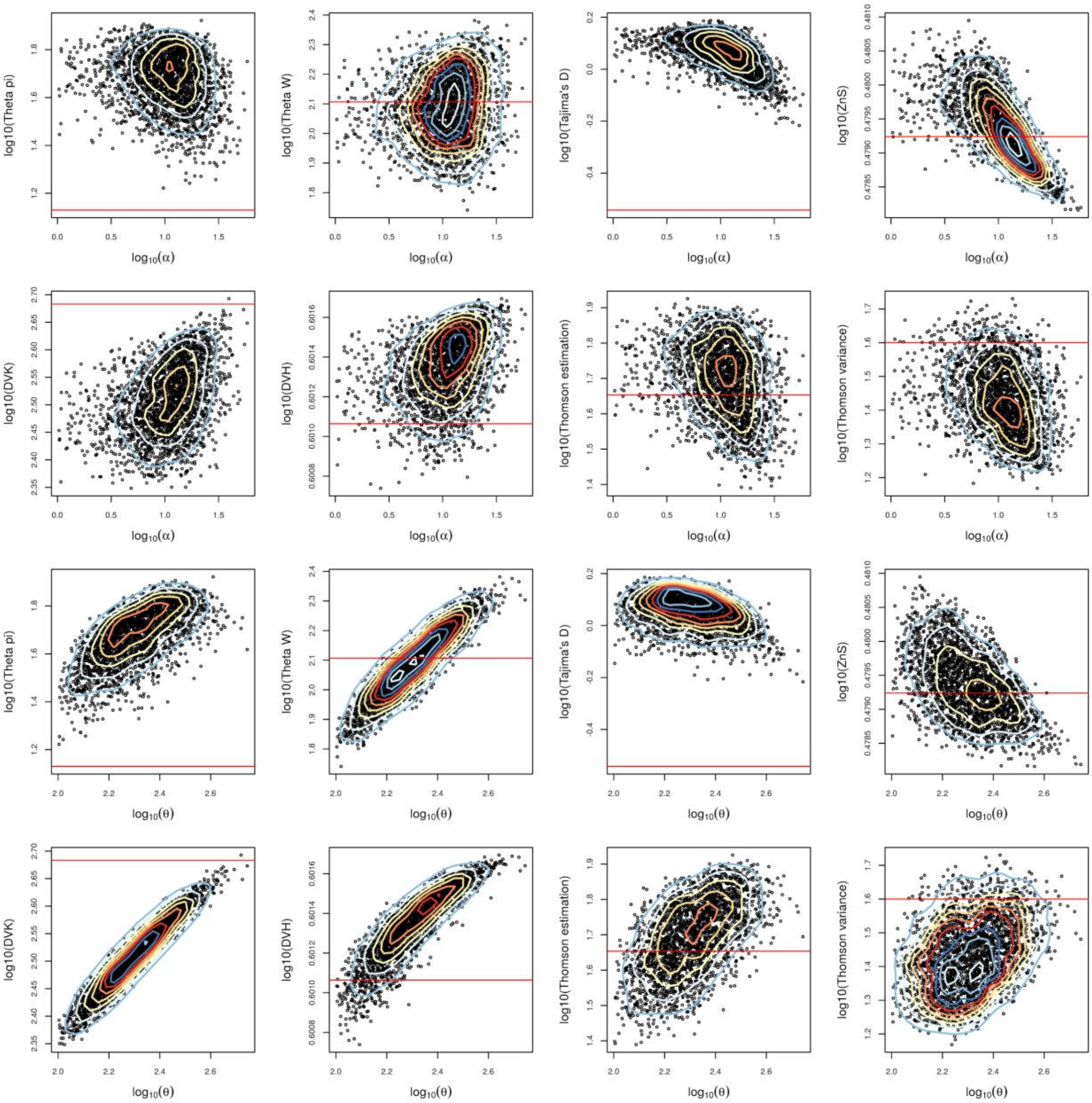

Figure S6: The space of the ABC accepted simulations of the European population. The contour lines on the scatterplot indicate the density estimation.

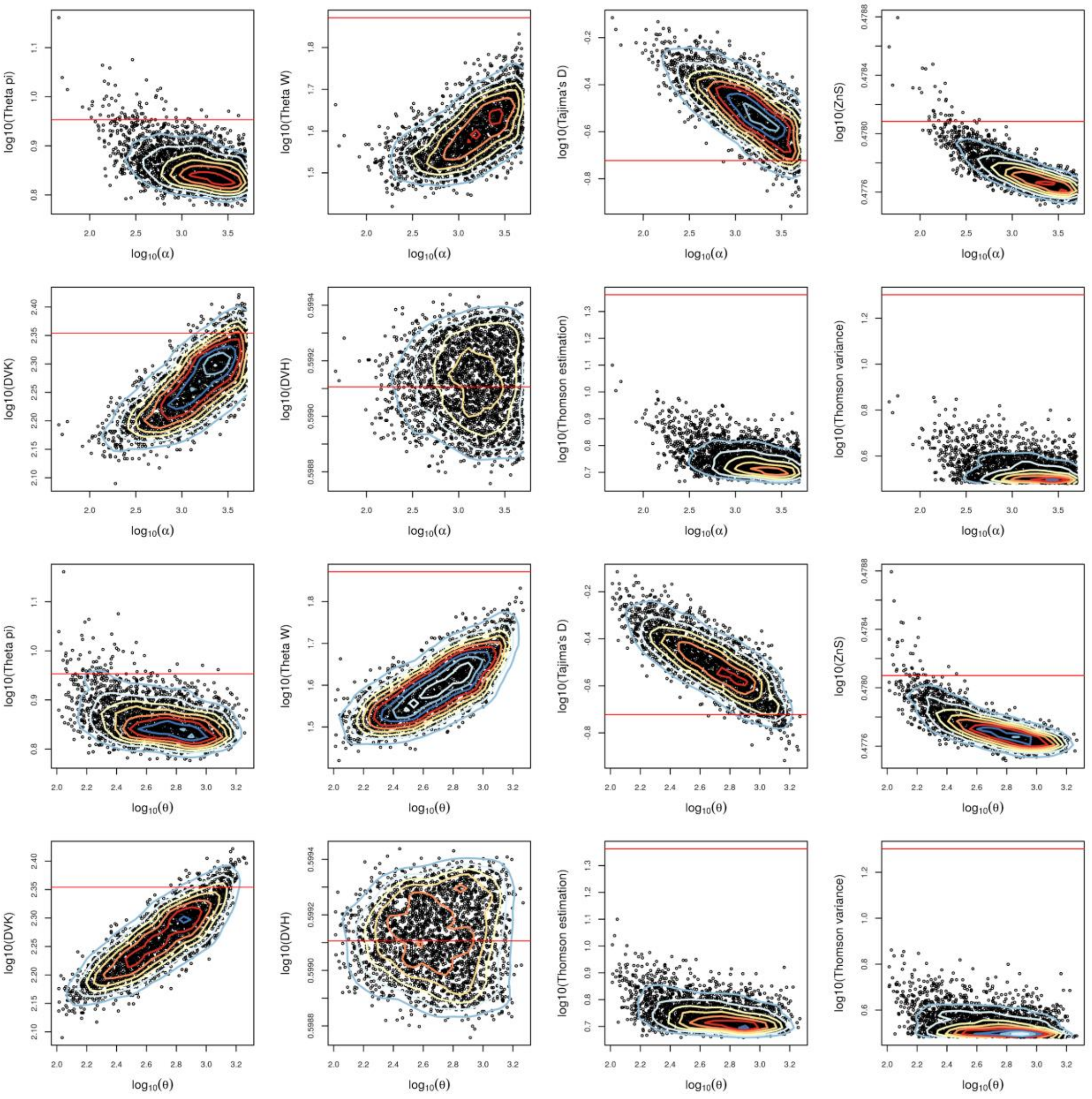

Figure S7: The space of the ABC accepted simulations of the Asian population. The contour lines on the scatterplot indicate the density estimation.

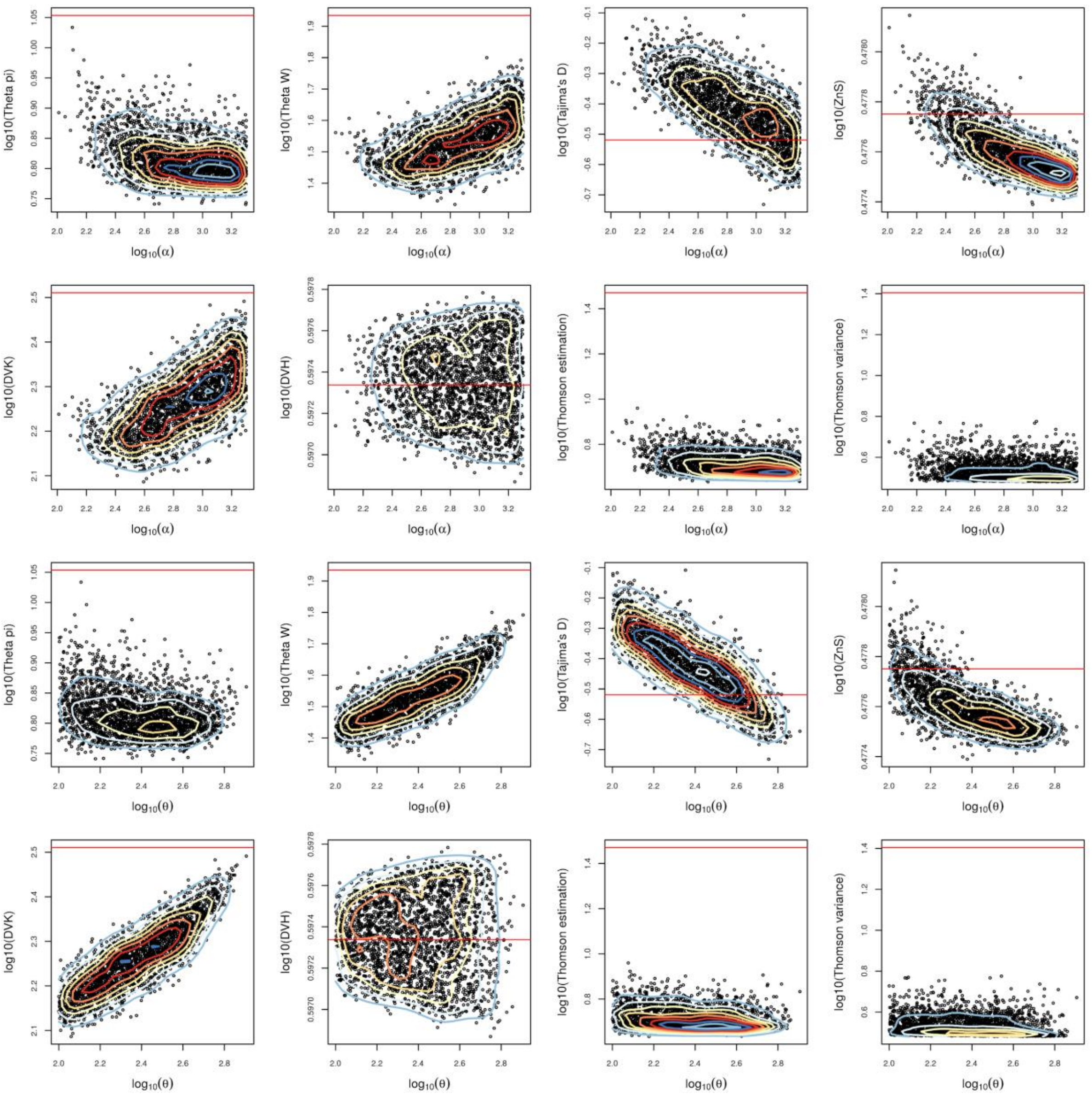

Figure S8: The space of the ABC accepted simulations of the Norhtern American population. The contour lines on the scatterplot indicate the density estimation.

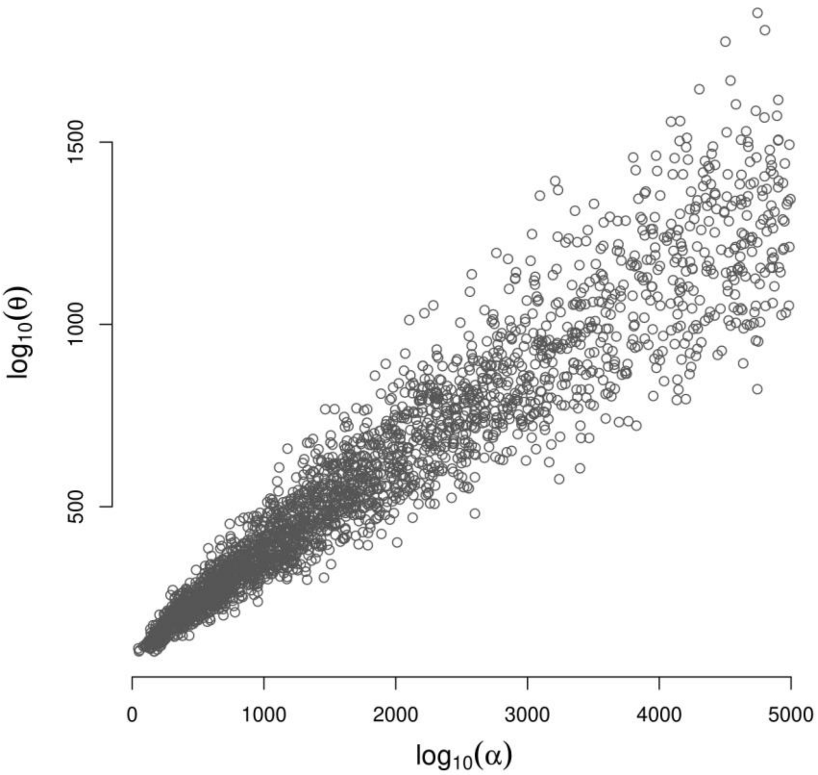

Figure S9: Correlation of Asia's population α and θ parameters of the ABC accepted simulations (unadjusted values). The Pearson correlation coefficient is 0.95.

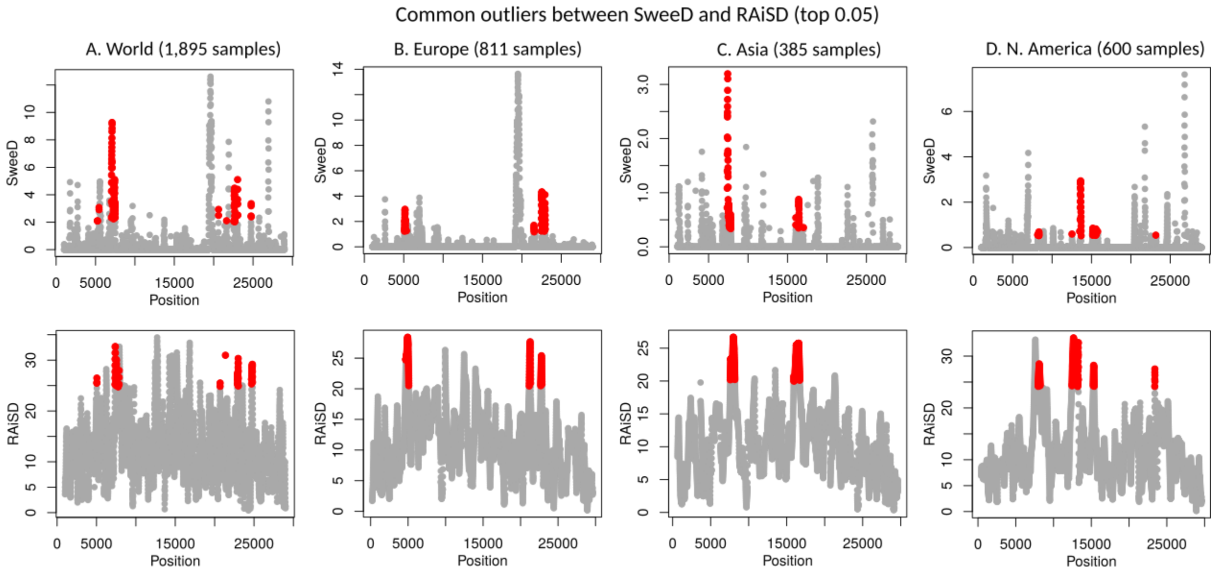

Figure S10: Common outliers between SweeD and RAiSD (top 0.05): A. World sample (1,895 genomes), B. Europe (811 genomes), C. Asia (385 genomes), and D. North America (600 genomes).
